# Exposure to Umbelliferone Reduces *Ralstonia solanacearum* Biofilm Formation, Transcription of Type III Secretion System Regulators and Effectors and Virulence on Tobacco

**DOI:** 10.3389/fmicb.2017.01234

**Published:** 2017-06-30

**Authors:** Liang Yang, Shili Li, Xiyun Qin, Gaofei Jiang, Juanni Chen, Bide Li, Xiaoyuan Yao, Peibo Liang, Yong Zhang, Wei Ding

**Affiliations:** ^1^Laboratory of Natural Products Pesticides, College of Plant Protection, Southwest UniversityChongqing, China; ^2^Yunnan Academy of Tobacco Agricultural ResearchYuxi, China; ^3^Laboratoire des Interactions Plantes-Microorganismes, UMR441, Institut National de la Recherche AgronomiqueCastanet-Tolosan, France; ^4^College of Resources and Environment, Southwest UniversityChongqing, China

**Keywords:** *R. solanacearum*, type III secretion system, biofilm formation, coumarins, umbelliferone, inhibitor, bacterial wilt

## Abstract

*Ralstonia solanacearum* is one of the most devastating phytopathogens and causes bacterial wilt, which leads to severe economic loss due to its worldwide distribution and broad host range. Certain plant-derived compounds (PDCs) can impair bacterial virulence by suppressing pathogenic factors of *R. solanacearum*. However, the inhibitory mechanisms of PDCs in bacterial virulence remain largely unknown. In this study, we screened a library of coumarins and derivatives, natural PDCs with fused benzene and α-pyrone rings, for their effects on expression of the type III secretion system (T3SS) of *R. solanacearum*. Here, we show that umbelliferone (UM), a 7-hydroxycoumarin, suppressed T3SS regulator gene expression through *HrpG–HrpB* and *PrhG–HrpB* pathways. UM decreased gene expression of six type III effectors (*RipX*, *RipD*, *RipP1*, *RipR*, *RipTAL*, and *RipW*) of 10 representative effector genes but did not alter T2SS expression. In addition, biofilm formation of *R. solanacearum* was significantly reduced by UM, though swimming activity was not affected. We then observed that UM suppressed the wilting disease process by reducing colonization and proliferation in tobacco roots and stems. In summary, the findings reveal that UM may serve as a plant-derived inhibitor to manipulate *R. solanacearum* T3SS and biofilm formation, providing proof of concept that these key virulence factors are potential targets for the integrated control of bacterial wilt.

## Introduction

*Ralstonia solanacearum*, the causal agent of bacterial wilt, is one of the most devastating bacteria among the top 10 plant pathogens (Mansfield et al., 2012). *R. solanacearum* is a Gram-negative soil-borne bacterium that infects more than 250 plant species, invading its host from the soil through root openings and colonizing the root cortex. It rapidly reaches the xylem vessels where it grows to high cell densities, resulting in host wilting and death (González and Allen, 2003; Liu et al., 2005; Genin, 2010).

During the infection process, *R. solanacearum* utilizes many different virulence factors to cause disease in susceptible hosts, including the type III secretion system (T3SS), extracellular polysaccharides (EPS), extracellular proteins, motility activity, and biofilm formation (Saile et al., 1997; Tans-Kersten et al., 2001; Liu et al., 2005; Yao and Allen, 2007; Poueymiro and Genin, 2009). The main pathogenicity determinant in *R. solanacearum* is T3SS (Coll and Valls, 2013), which it deploys to secrete proteins directly inside the plant cell. These proteins, called type III effectors (T3Es), interact with molecules to manipulate plant cellular function, suppressing immunity and inducing the pathogen to multiply and spread (Macho, 2016; Mukaihara et al., 2016). Recently, many studies have demonstrated that expression of T3SS-associated genes is regulated by environmental factors, such as pH, growth phase, temperature, nutrition, or cell density (Arlat et al., 1992; Wei et al., 1992; Van Dijk et al., 1999; Tang et al., 2006; Stauber et al., 2012). T3SS-associated genes are also regulated by natural-derived compounds or chemically synthesized compounds (Felise et al., 2008; Yang et al., 2008, 2014; Aiello et al., 2010; Duncan et al., 2012; Wu et al., 2015), making T3SS a particularly appealing target for the development of new agents for disease control. Because antimicrobial agents that utilize T3SS-specific inhibitors would affect pathogen virulence rather than viability, T3SS is also an attractive target for antimicrobial agents that generate low selective pressure for antimicrobial resistance development (Escaich, 2008; Rasko and Sperandio, 2010). Recently, certain plant phenolic compounds and their derivatives were found to inhibit T3SS in the plant pathogens *Erwinia amylovora* and *Dickeya dadantii* (Li et al., 2009, 2015; Khokhani et al., 2013). *HrpB* and *ExsA*, AraC class regulators, are important components of T3SS in bacteria that infect plants or animals. Recent study has identified T3SS inhibitors like *N*-hydroxybenzimidazoles inhibits ExsA-dependent T3SS gene expression by interacting with the carboxy-terminal domain of ExsA (Marsden et al., 2016). As mentioned above, plant-derived compounds (PDCs) that inhibit T3SS expression in plant pathogens have recently been attracting increasing attention due to their potential abundant sources and environmental safety. Nonetheless, effective inhibitors need to be identified, and the regulation pathway of T3SS inhibitors remains largely uncharacterized.

Evidence to date suggests that *R. solanacearum* has evolved the ability to manipulate plant cell release or to degrade plant compounds, including galacturonic acid released by extracellular polygalacturonases from the plant cell walls, nourishing the bacteria during pathogenesis and with rapid disease onset (Allen et al., 1991; González and Allen, 2003). *R. solanacearum* is also proven to degrade plant salicylic acid (SA) to protect itself from inhibitory levels of this compound and also to enhance its virulence on the plant hosts like tobacco that use SA as a defense signal molecule (Lowe-Power et al., 2016). It has been observed that an exogenous compound could induce tomato resistance against *R. solanacearum* via overexpression of ethylene and jasmonic acid (Ghareeb et al., 2011). The PDCs sclareol and *cis*-abienol are isolated from tobacco as inducers of *R. solanacearum* resistance (Seo et al., 2012). PDCs play an important role in inducing plant resistance to prevent *R. solanacearum* invasion, also play an important role in the interaction between the pathogen and host. However, the underlying mechanisms of PDCs such as coumarins on the virulence factors of plant pathogens remain unknown.

Coumarins, naturally derived compounds composed of fused benzene and α-pyrone rings, have been shown to possess many biological activities, such as antibacterial, antifungal, anticoagulant, antioxidant, anticancer, and anti-inflammatory activity (Barot et al., 2015; Yang et al., 2016). Due to specific structural characteristics, some coumarins are regarded as phytoalexins biosynthesized by plant tissues in response to pathogenic infection that play a role in disease resistance (Andreae, 1948). Scopoletin is a phenolic coumarin and an important member of the group of phytoalexins isolated from many plants (Tal and Robeson, 1986). Umbelliferone (UM, 7-hydroxycoumarin) is a phytoalexin found in the roots of the sweet potato (Minamikawa et al., 1963) and *Pharbitis nil* (Yaoya et al., 2014). Plant-associated bacteria can metabolize UM (Parales and Harwood, 2002), and our previous studies indicated that hydroxycoumarins such as UM and daphnetin exert antibacterial activities against *R. solanacearum* and suppress expression of the T3SS-associated gene *HrpG* (Yang et al., 2016). As mentioned above, these results highlight the importance of investigating coumarins regulatory mechanisms in inhibition of T3SS expression in *R. solanacearum*.

Some PDCs can impair bacterial virulence by suppressing pathogenic factors of *R. solanacearum*. We hypothesized that coumarins, as specific PDCs, may alter expression of *R. solanacearum* pathogenic factor T3SS. In this study, a library of coumarins was screened for their effect on T3SS expression in *R. solanacearum*. We used quantitative real-time polymerase chain reaction (qRT-PCR) to investigate the molecular mechanism of UM, one of the best T3SS inhibitors, with regard to T3SS and T2SS gene expression in *R. solanacearum*. Furthermore, the effect of UM on the bacterial population in roots and stems and suppression of disease development in tobacco were investigated.

## Materials and Methods

### Materials and Bacterial Strains

The bacterial wilt pathogen *R. solanacearum* CQPS-1 (phylotype I, race 1, biovar 3) and the *RipX-lacZYA* reporter strain CQPS-1 were used in this study. The reporter strain was constructed using the recombinant plasmid ppop3 as previously described (Zhang et al., 2011). *R. solanacearum* was incubated at 28°C in rich B medium or hydroponic plant culture medium supplemented with 2% sucrose [plant-sucrose (PS) medium; Yoshimochi et al., 2009].

The coumarins (HPLC ≥ 98%) used in the study were purchased from Shanghai Yuanye Bio-Technology Co., Ltd. (Shanghai, China) and Adamas Reagent, Ltd. (Shanghai, China). Each compound was dissolved in dimethyl sulfoxide (DMSO) to a final concentration of 10 mg/mL, and the compound solvent was added to the rich B or PS medium to prepare different concentrations of compound suspensions.

### β-Galactosidase Assay

The effect of coumarins on expression of *RipX* was determined by measuring β-galactosidase activity of *lac-ZYA* reporter gene as previously described, with minor modifications (Zhang et al., 2011). The *RipX-lacZYA* reporter strain was inoculated in rich B medium for 6–7 h with shaking at 28°C, and bacterial cells were collected. The bacterial suspension was transferred to PS medium supplemented with DMSO or 50 mg/L coumarins. When the OD_600_ of the bacterial suspension reached 0.1–0.2, the β-galactosidase activity was measured. The enzyme assay was repeated two times.

### RNA Isolation and Quantitative Real-Time PCR

Total RNA extraction and quantitative real-time PCR (qRT-PCR) were performed as previously described (Wu et al., 2015). For bacterial RNA analysis, an overnight-cultured *R. solanacearum* suspension was inoculated in PS medium supplemented with UM or DMSO and then incubated at 28°C with shaking at 180 rpm for 6–7 h. The samples were centrifuged at 5000 rpm for 10 min at 4°C, and the supernatant was removed, the treated bacterial cells were collected. One microgram of cDNA was synthesized in a 20-μL reaction mixture using the iScript cDNA synthesis kit (Bio-Rad, Hercules, CA, United States). The primers used for the tested genes were synthesized by BGI Technologies (Shenzhen, Guangzhou, China). All quantitative real-time PCR analyses were carried out in 96-well plates in a 20-μL reaction system. Three technical replicate reactions were used for each sample. Normalized gene expression was calculated by Bio-Rad CFX, and *SerC* was used as a reference gene (Monteiro et al., 2012). All assays were performed three times in biological repeats.

### Biofilm Assay

Biofilm formation of *R. solanacearum* supplemented with UM was performed in 96-well polystyrene microtiter plates as previously reported (Yao and Allen, 2007). Briefly, an overnight-cultured *R. solanacearum* suspension was inoculated in B medium supplemented with DMSO or different concentrations of UM ranging from 6.25 to 50 mg L^-1^. The samples were incubated at 30°C without shaking for 24 and 32 h. Biofilms were stained with crystal violet, dissolved in 95% ethanol and quantified by absorbance at 530 nm (OD_530_). All assays were carried out at least three times in biological repeats.

### Swimming Motility Assay

The swimming motility of *R. solanacearum* was assessed on semi-solid motility media as previously reported (Kelman and Hruschka, 1973). Different concentrations of UM and DMSO were added to the semi-solid motility medium, and a 3-μL overnight-cultured *R. solanacearum* suspension was dropped on the center of the plate. White halos were measured after 48 h cultivation at 30 ± 1°C, and motility was measured as the halo diameter. All assays were carried out in triplicate.

### Virulence Assay

The naturalistic soil soak assay was used to evaluate the virulence of *R. solanacearum* after UM treatment. Briefly, unwound 6-week-old tobacco plants (Yunyan 87) were soaked in 25 or 50 mg L^-1^ UM or DMSO. Individual plants were inoculated by pouring 20 mL of bacterial suspension into the soil to create a final inoculation density of 1 × 10^8^ CFU/g soil. Inoculated plants were placed into a climate room at 28°C with a 14 h/10 h light/dark cycle. Symptoms for each plant were scored daily, using a disease index scale from 0 to 4 (0 indicated no symptoms; 1 indicated 1–25% of leaves wilted; 2 indicated 26–50% of leaves wilted; 3 indicated 51–75% of leaves wilted; 4 indicated 76–100% of leaves wilted). Each treatment contained 16 plants in an independent experiment, and the inoculation assay was repeated three times.

The water-inoculation assay was used to evaluate the effect of UM treatment on the population of *R. solanacearum* in tobacco roots. We chose 4-week-old tobacco plants, cleared the medium and placed the tobacco root into different concentrations of UM suspension for 5 min. The plants were then placed into MS liquid medium supplemented with a 0.5% *R. solanacearum* suspension. After four dips, the roots were collected, the root suspension was diluted from 10^-1^ to 10^4^, and 100 μL was placed onto SMSA medium to quantify CFU.

To determine the bacterial population size in tobacco stems, 200 mg of tissue at the base of the stems was destructively harvested. The tissue was ground in water and dilution plated onto SMSA medium for CFU quantification. Each treatment had two replications, and the entire experiment was performed three times. The semi-selective SMSA medium used in the assay was previously described (Elphinstone et al., 1996).

## Results

### The Effect of Coumarins on Expression of *RipX* in *R. solanacearum*

**FIGURE 1:**
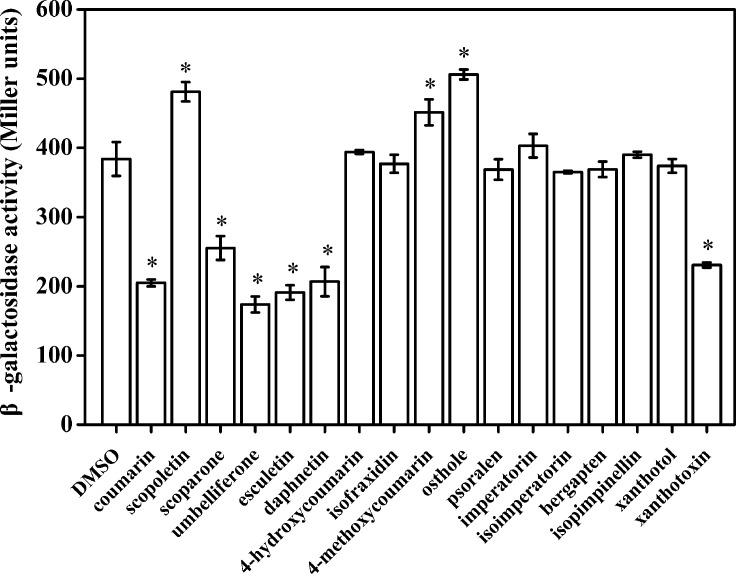
The effect of coumarins on expression of RipX in *R. solanacearum*. Expression of *R. solanacearum* RipX was measured as β-galactosidase activity of the lacZYA reporter fusion gene in PS medium or PS medium supplemented with 50 mg/L of different coumarins. *R. solanacearum* was incubated in PS medium for 6–7 h. Once the OD_600_ of bacterial suspension was 0.1–0.2, the promoter activities were determined. The coumarins were assayed two times, and error bars indicate the standard deviation. Asterisks indicate significant differences compared with DMSO treatment (*P* < 0.05, Student’s *t*-test).

Our primary research indicated that hydroxycoumarins such as UM and daphnetin have the potential to act as T3SS inhibitors because they significantly repress expression of *HrpG* (Yang et al., 2016). These results suggested that coumarins may inhibit expression of T3SS regulators and T3Es in *R. solanacearum*. To screen for coumarins that affect T3Es expression of in *R. solanacearum*, the *RipX*-lacZYA reporter strain was constructed for assessing β-galactosidase activity. Among the screened 17 plant-derived coumarins, (**Supplementary Figure S1**) six coumarins (coumarin, scoparone, UM, esculetin, daphnetin, and xanthotoxin) significantly inhibited *RipX* promoter activity (**Figure 1**). Compared with DMSO treatment, UM was the best inhibitor, with a 2.2-fold decrease in β-galactosidase activity. Therefore, UM was used for investigating mechanisms related to T3SS regulators and T3Es of *R. solanacearum*.

### The Expression of *RipX* Is Inhibited by UM

**FIGURE 2:**
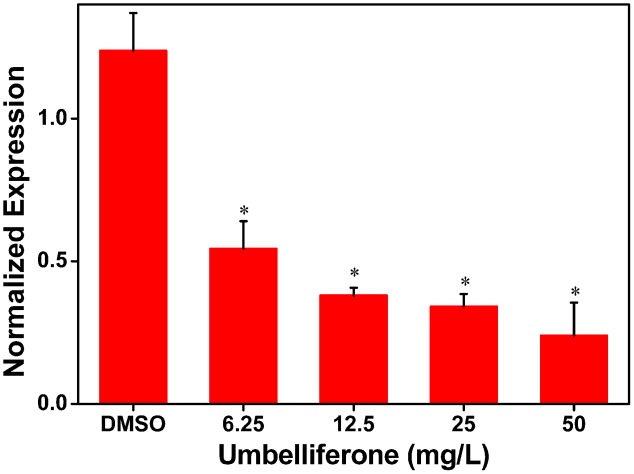
UM inhibits expression of the type III representative effector *RipX* in *R. solanacearum* in a concentration-dependent manner. *R. solanacearum* cells incubated in PS medium were supplemented with different concentrations of UM, ranging from 6.25 to 50 mg L^-1^. After incubated 6–7 h, *RipX* expression of *R. solanacearum* was measures as the normalized expression using qRT-PCR. The ΔΔCq method was used to normalize the gene expression, and *SerC* was used as the reference gene. UM treatment inhibits expression of *RipX* two- to fourfold. Three biological replicates were performed, and error bars indicate the standard deviation. Asterisks indicate statistically differences between DMSO treatment and UM treatment (*P* < 0.05, Student’s *t*-test).

To further determine the inhibitory activity of UM on *RipX* expression, qRT-PCR was performed to investigate *R. solanacearum* expression at the transcriptional level in samples supplemented with different concentrations of UM, ranging from 6.25 to 50 mg/L. Compared to the DMSO control, a significantly lower expression level of *RipX* mRNA was observed when the PS medium was supplemented with different concentrations of UM (**Figure 2**). At 50 mg L^-1^ of UM treatment, the transcription level was reduced by 80.67% compared to the DMSO control. The reduction in *RipX* expression in samples supplemented with 25, 12.5, and 6.25 mg L^-1^ UM was 71.42, 69.30, and 56.06%, respectively. These results demonstrate that the inhibitory activity of UM on transcriptional expression of *RipX* occurred in a concentration-dependent manner.

### UM Inhibits Expression of T3SS Regulators through the PrhG–HrpB and HrpG–HrpB Pathways

**FIGURE 3:**
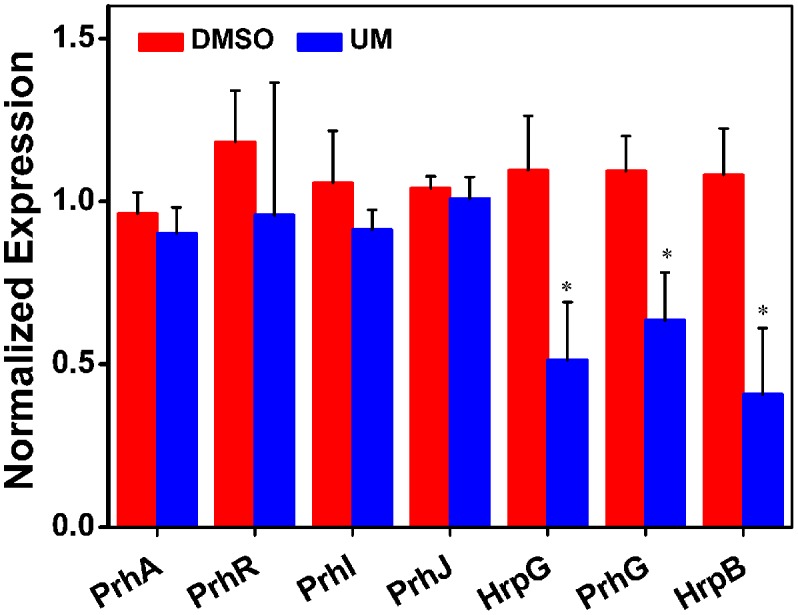
The effect of UM on expression of T3SS pathway genes. Inhibition of *R. solanacearum* T3SS by UM occurs through the *HrpG–HrpB* and *PrhG–HrpB* pathways. Expression of T3SS pathway genes was evaluated by qRT-PCR in PS medium supplemented with DMSO or 50 mg L^-1^ UM. *SerC* was used as the reference gene to normalize gene expression using the ∆∆Cq method. The results reflect three biological replicates, and error bars indicate the standard deviation. Asterisks indicate statistically differences in gene expression of bacterial cells supplemented with DMSO or 50 mg L^-1^ UM (*P* < 0.05, Student’s *t*-test).

*RipX* is a T3E gene, its expression is directly controlled by *HrpB*, the expression of which is further regulated by *HrpG*, *PrhG* and other upstream regulators. As the suppression effect of UM on *RipX* expression was validated, we sought to determine how UM affects the regulatory components of other T3SS regulators, which have been well described in *R. solanacearum* (Peeters et al., 2013). To confirm this finding, mRNA levels of T3SS regulators were measured after DMSO or UM treatment. Our results showed that expression of some T3SS upstream regulators was significantly reduced, such as *HrpG*, *PrhG*, and *HrpB*, compared to the DMSO control (**Figure 3**). However, UM had no effect on expression of other T3SS upstream regulators (*PrhA*, *PrhR*, *PrhI*, and *PrhJ*). UM-mediated regulation of *R. solanacearum* T3SS regulators mainly occurred through the *HrpG–HrpB* or *PrhG–HrpB* pathway.

### UM Suppresses Expression of Most Tested Type III Effector Genes

**FIGURE 4:**
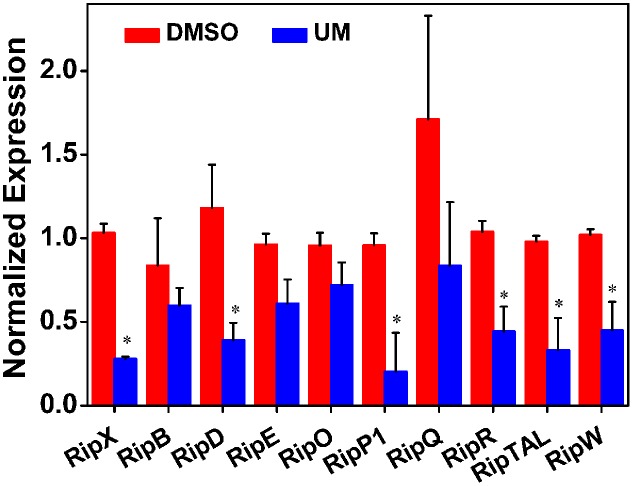
Effect of UM on the inhibition of 10 representative type III effector genes in *R. solanacearum* cells. Expression of type III effector genes was measured by qRT-PCR in PS medium supplemented with DMSO or 50 mg L^-1^ UM. *SerC* was used as reference gene to normalize the type III effector genes expression using the ∆∆Cq method. The results reflect three biological replicates and error bars indicate the standard deviation. Asterisks means the type III effector genes are significantly inhibited by UM treatment compared with DMSO treatment (*P* < 0.05, Student’s *t*-test).

In *R. solanacearum*, *HrpB* is a downstream regulator in T3SS signal cascade and directly controls transcription of T3E genes. Based on initial experiments, we found that UM suppressed expression of both T3SS downstream regulatory gene *hrpB* and the T3E gene *RipX*. To determine whether the suppression of *hrpB* by UM results in transcriptional activation of other effector genes, qRT-PCR was performed to examine the mRNA levels of other effector genes in the presence and absence of UM. Because *R. solanacearum* has a large repertoire of effectors and it is rather difficult to evaluate the expression of all other effector genes, we chose 10 representative effector genes for this experiment. Based on the results, many of the tested T3Es genes were significantly suppressed by UM treatment, including *RipX*, *RipD*, *RipP1*, *RipR*, *RipTAL*, and *RipW* (**Figure 4**). Compared with DMSO treatment, the mRNA levels of T3Es genes, especially *RipX*, *RipD*, and *RipP1*, were significantly decreased three- to fourfold under UM treatment. In contrast, a few T3E genes, including *RipB*, *RipE*, *RipO*, and *RipQ*, were similarly expressed in DMSO- or UM-treated cells. The results indicated that many other T3E genes may be suppressed by T3SS inhibitor UM.

**FIGURE 5:**
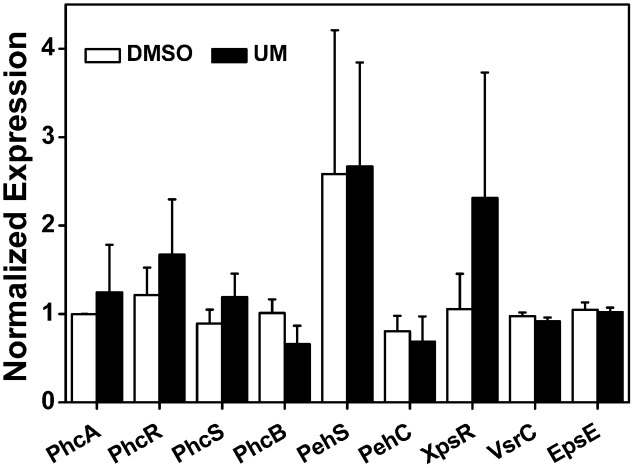
Effect of UM on expression of other virulence-associated genes in *R. solanacearum*. qRT-PCR was performed to measure the relative expression level of virulence-associated genes in PS medium supplemented with DMSO or 50 mg L^-1^ UM. The assay was performed three biological replicates (*P* < 0.05, Student’s *t*-test).

To further evaluate the effect of UM on other virulence factors that play important roles at different infection stages, we measured the mRNA levels of the following: the quorum sensing system and the type II secretion system *PhcA*; the quorum sensing regulators *PhcB*, *PhcR*, *PhcS*, *PehS*, and *PehC*; the EPS secretion-related genes *XpsR* and *EpsE*; and the swimming regulator gene *VsrC*. As shown in **Figure 5**, the nine tested virulence factor genes showed no significant differences between UM and DMSO treatments (*P* < 0.05). It appears that UM does not affect expression of most other virulence regulation genes.

### UM Reduces the Biofilm Formation of *R. solanacearum*

**FIGURE 6:**
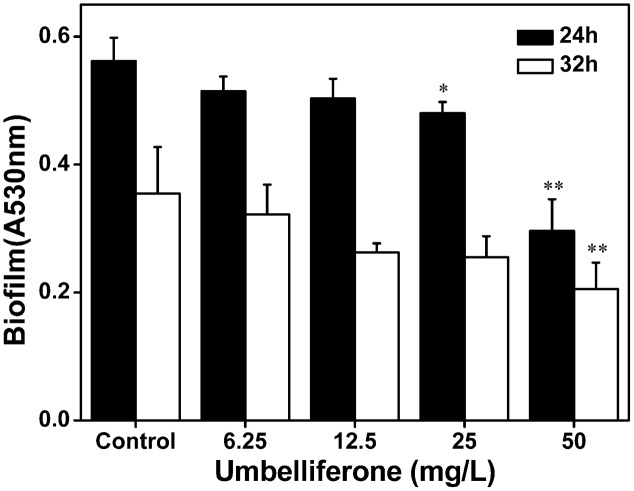
Effect of UM on biofilm formation of *R. solanacearum* ranging from 6.25 to 50 mg L^-1^. Data shown are the means of independent experiments with a least three replicates. Bars represent standard errors of the means. Statistical significance was determined by Student’s *t*-test in comparison with the DMSO control treatment (^∗^*P* < 0.05, ^∗∗^*P* < 0.01).

To evaluate biofilm formation by *R. solanacearum* supplemented with UM, we used a standard polyvinyl chloride (PVC) microtiter plate assay using concentrations ranging from 6.25 to 50 mg L^-1^. As shown in **Figure 6**, UM treatment 50 mg L^-1^ significantly reduced biofilm formation by *R. solanacearum* (P < 0.05). Compared with the control treatment, biofilm formation at 50 mg L^-1^ UM was also significantly reduced by 47.28 and 42.08% at 24 and 32 h, respectively. The inhibitory activity of UM was concentration dependent. As the motility of *R. solanacearum* plays an important role in biofilm formation; the swimming motility of cells supplemented with UM was evaluated on semi-solid motility agar. After 2 days at 30°C, motile colonies of *R. solanacearum* supplemented with UM at concentrations ranging from 6.25 to 50 mg L^-1^ were surrounded by a white halo with radiating streaks, producing swimming haloes similar to those the control treatment (**Supplementary Figure S2**), with no significant differences.

### UM Reduces the Virulence of *Ralstonia solanacearum* in Tobacco Plants

**FIGURE 7:**
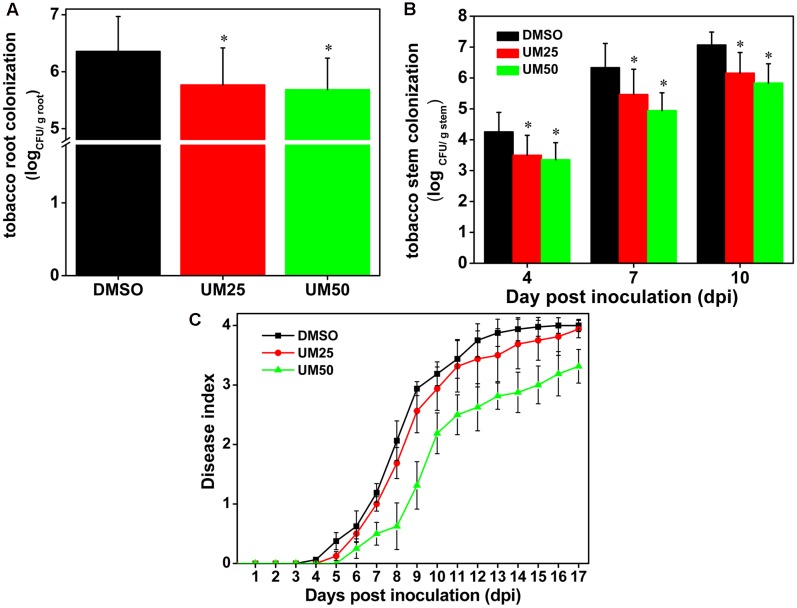
UM treatment reduces virulence of *R. solanacearum* in tobacco. **(A)** Bacterial density in root tissue was quantified by dilution plating roots from water-inoculated plants. Asterisks indicate *P* < 0.05 (Student’s *t*-test). **(B)** *R. solanacearum* density in the stem was quantified by dilution tissue suspension from 10^-1^ to 10^-4^, with 100 μL stem tissue suspension on SMSA medium. The plates were incubated in 30°C for 2 days, and the bacterial abundance was quantified in the plates. Each treatment included 10 plants, and the assay included three biological replicates. Asterisks indicate significant differences between the DMSO control and UM treatment (*P* < 0.05). **(C)** Effect of UM on the disease index of bacterial wilt in tobacco. Unwounded tobacco plants were soil-soak inoculated at a concentration of 20 mL (1 × 10^8^ CFU/mL) and incubated at 30 ± 1°C under a 14-h/10-h light/dark cycle.

Based on the strong suppressive activity of UM against biofilm formation and expression of T3SS regulators and T3Es genes in *R. solanacearum*, the effect of UM on bacterial wilt disease progression was evaluated. As shown in **Figure 7C**, compared with DMSO, UM at 50 mg L^-1^ altered the disease progress of bacterial wilt (*P* < 0.05). These findings suggest that UM reduces *R. solanacearum* virulence in tobacco plant by suppressing biofilm formation, expression of T3SS regulators and T3Es genes.

We further investigated the effects of UM on bacterial populations in tobacco roots and stems. As shown in **Figure 7A**, UM treatment significantly reduced *R. solanacearum* populations in tobacco roots in a concentration-dependent manner after incubated four dip in water inoculation assay. In addition, 50 mg L^-1^ UM significantly reduced the population at the base of the tobacco plant stem. Compared with DMSO treatment, the pathogen populations of tobacco stems supplemented with 50 mg L^-1^ UM were significantly reduced, by 21.18, 21.96, and 17.45% at 4, 7, and 10 days after inoculation, respectively (**Figure 7B**).

## Discussion

As a landmark discovery, advances in the study of bacterial virulence factors have provided evidence that T3SS is one of the main pathogenicity determinants in *R. solanacearum* (Peeters et al., 2013). In the present study, 17 coumarins were evaluated for their ability to inhibit or induce expression of the *R. solanacearum* T3Es gene *RipX* (**Figure 1**). Our experiment revealed that six coumarins (coumarin, scoparone, UM, esculetin, daphnetin, and xanthotoxin) significantly inhibited expression of *RipX* and that three coumarins (scopoletin, 4-methoxycoumarin, and osthole) significantly induced *RipX* expression. We then chose a best T3SS inhibitor (UM) to investigate the mechanism of *R. solanacearum* T3SS regulation by PDCs. Further experiments demonstrated that UM suppresses expression of T3SS regulators through the *PrhG–HrpB* and *HrpG–HrpB* pathways and inhibits expression of many T3Es genes (**Figures 3**, **4**). Furthermore, UM suppressed biofilm formation without affecting swimming activity, and bacterial populations of *R. solanacearum* were reduced by UM treatment in the roots and stems of tobacco. In addition, UM reduced the virulence of *R. solanacearum* by suppressing biofilm formation as well as, expression of T3SS regulators and T3Es genes, resulting in delayed tobacco bacterial wilt disease progression (**Figure 7**).

Much evidence has suggested that screen compounds to target virulence factors is an effective strategy for controlling bacterial disease (Rasko and Sperandio, 2010; Wu et al., 2015). High-throughput screening is a powerful tool for identifying small molecule inhibitors that suppress expression of T3SS regulators (Pan et al., 2007). Recent studies have identified several classes of PDCs, as well as synthetic compounds as active T3SS inhibitors in a wide range of Gram-negative bacterial pathogens, including *R. solanacearum*, *E. amylovora*, *D. dadantii*, *Xanthomonas oryzae*, and *Yersinia pestis* (Jessen et al., 2014; Yang et al., 2014; Li et al., 2015; Lowe-Power et al., 2016). These PDCs determined to act as T3SS inhibitors include SA, *p*-coumaric acid, *trans*-4-hydroxycinnamohydroxamic acid, 4-methoxycinnamic acid, benzoic acid, *trans*-2-phenylcyclopropane-1-carboxylic acid, and *N*-(4-methoxycinnamyl)phthalimide (Li et al., 2009, 2015; Khokhani et al., 2013; Fan et al., 2017). In our study, six coumarins (coumarin, scoparone, UM, esculetin, daphnetin, and xanthotoxin) were found to significantly inhibit expression of the T3Es gene *RipX* (**Figure 1**), showing proof of concept that expression of T3SS in diverse bacterial pathogens can be inhibited by PDCs. Interestingly, T3SS expression in *R. solanacearum* and other bacteria can also be induced by certain PDCs, such as oleanolic acid, chlorogenic acid, scopoletin, taxifolin, *o*-coumaric acid, and *trans-*cinnamic acid (Yang et al., 2008; Wu et al., 2015). These studies suggest that PDCs may induce expression of T3SS in diverse bacterial pathogens, which is consistent with our finding that the *R. solanacearum* T3Es genes *RipX* was induced by some tested coumarins.

UM (7-hydroxycoumarin) is a coumarins compound distributed in a variety of plant species and has several promising biological activities, including anticoagulant, antioxidant, antibacterial, and antifungal (Barot et al., 2015). Our previous study proved that UM possesses antibacterial activity against *R. solanacearum*, with an IC_50_ of 96.88 mg L^-1^ when incubated for 24 h (Yang et al., 2016). It is interesting that UM was able to inhibit expression of T3SS regulator and T3Es genes in this study, expanding the biological functions of this special compound in plants. The T3SS regulator inhibitory activity of UM may be related to its specific structural characteristics. Previously, it has been demonstrated that plant phenolic compounds can serve as T3SS inhibitors or inducers (Khokhani et al., 2013), suggesting that different substitutions may be responsible for the same type of compounds exhibiting distinct T3SS activities. A strict requirement for the *R-enantiomer* at its stereocenter and tolerance for a variety of substituents on one of its two aromatic rings are key factors for the ability of a phenoxyacetamide series to function as T3SS inhibitors (Aiello et al., 2010). The *para* positioning of the hydroxyl group in the phenyl and the double bond of *p*-coumaric acid may be important for its T3SS inhibition activity (Li et al., 2009). Which is consistent with our finding that coumarins can act as T3SS inhibitors or inducers. Further analysis of several coumarins suggested that the hydroxyl-substituents of coumarins might be important for inhibiting expression of T3SS regulator and T3Es genes.

**FIGURE 8:**
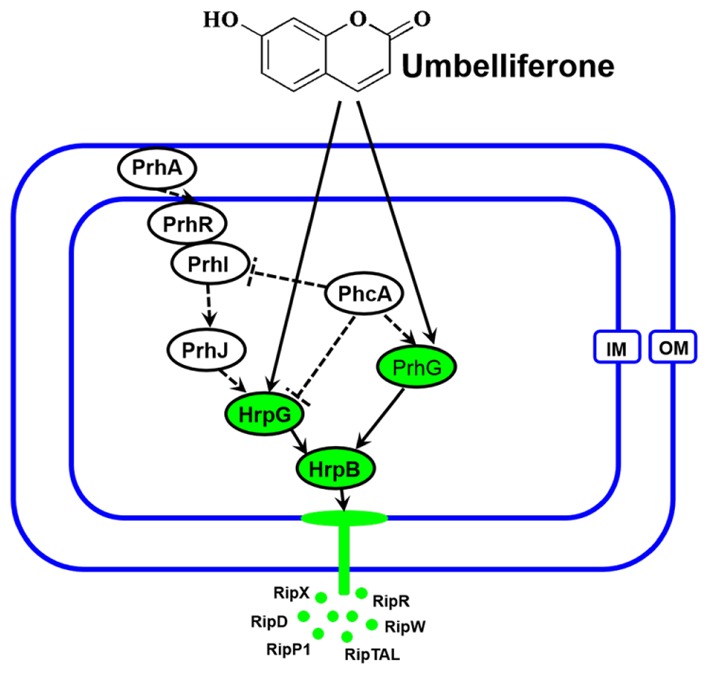
Mode of UM in inhibiting *R. solanacearum* T3SS. The *hrp* gene expression in *R. solanacearum* is directly controlled by HrpB. HrpB is further controlled by two independent cascades involving PhcA–PrhG and PrhA–PrhR/PrhI–PrhJ–HrpG. The global regulator *PhcA* can also modulate T3SS expression through PrhI and HrpG (Peeters et al., 2013). In this study, we observed that T3SS inhibiter UM directly suppresses expression of *HrpG* and *PrhG*, inhibited the type III effector genes expression (e.g., *RipX*, *RipD*, *RipW, RipR*, *RipTAL*, and *RipP1*) through the *HrpG–HrpB* and *PrhG–HrpB* pathways. Ovals with green background indicate regulators inhibited by UM. Solid line arrows indicate direct induction of these regulators by UM. IM, inner membrane; OM, outer membrane.

The T3SS regulator components and signaling cascade of *R. solanacearum* have been well characterized (Marenda et al., 1998; Aldon et al., 2000). PrhA, an outer membrane receptor at the top of the *hrp* regulatory pathway, is responsible for perceiving plant signals and activating downstream regulators. In this study, we found that UM inhibited expression of the downstream T3SS regulator HrpG, but it did not affect expression of upstream regulators genes, such as *PrhA*, *PrhR*, *PrhI*, and *PrhJ* (**Figure 3**). Interestingly, HrpG activation of *R. solanacearum* cells was regulated only by the PrhA–PrhR–PrhI–PrhJ–HrpG pathway in a nutrient-rich medium. While, metabolic or plant signals affect the receptors on the outer membrane, activating the sensor kinase on the inner membrane and then activating the expression of *HrpG* in nutrient-poor medium or plant co-cultivation experiments (Yoshimochi et al., 2009). Consistent with this previous study, our results suggested that expression of the T3SS regulator HrpG was significantly inhibited by UM and *R. solanacearum* may recognized UM as a metabolic signal to directly target T3SS downstream regulator HrpG. The *hrp* gene expression in *R. solanacearum* cells is also controlled by another T3SS regulator, PrhG, which occurs an independent pathway (Plener et al., 2010; Zhang et al., 2013). This regulator controls expression of T3SS under medium conditions but not in the presence of plant cells (Plener et al., 2010). Interestingly, we found that the T3SS regulator PrhG was also inhibited by UM in this study. Together, our results suggest that T3SS inhibitor UM inhibited expression of T3SS regulators and T3Es genes through the *HrpG–HrpB* and *PrhG–HrpB* pathways (**Figure 8**).

Recent studies have demonstrated that *R. solanacearum* utilizes T3SS to secrete T3Es to accelerate bacterial wilt progression. T3Es interact with molecules to manipulate plant cellular function, suppressing immunity and inducing pathogen multiplication and spread (Macho, 2016). In this study, we found that UM significantly suppressed the expression of some tested T3Es genes (*RipX*, *RipD*, *RipP1*, *RipR*, *RipTAL*, and *RipW*; **Figure 4**). Previously, it was demonstrated that half of effector genes are up-regulated in wilting tomato plants compared to expression in rich medium (Jacobs et al., 2012). This indicates that T3Es production is required during stages of bacterial wilt. Similar to many plant pathogenic bacteria, *R. solanacearum* forms biofilms on plant roots, contributing to invasion and infection (Yao and Allen, 2007). Many studies have identified potential chemically synthesized or PDCs to target biofilm formation by pathogenic bacteria (O’Loughlin et al., 2013). In the current study, biofilm formation of *R. solanacearum* with 50 mg L^-1^ UM was significantly reduced by 47.28 and 42.08% at 24 and 32 h, respectively (**Figure 5**). An ensuing inoculation assay using tobacco showed that UM treatment significantly reduced the *R. solanacearum* population in the roots and stems, and altered bacterial wilt disease progression (**Figure 7**). The findings suggest that UM reduces the virulence of *R. solanacearum* by suppressing biofilm formation and transcriptional expression of certain effector genes.

In summary, UM significantly reduced the biofilm formation by *R. solanacearum* without affecting swimming activity. The pathway by which UM regulates T3SS expression in *R. solanacearum* was first investigated. UM suppressed expression of T3SS regulators through the HrpG–HrpB and PrhG–HrpB pathways. In addition, T3Es genes *RipX*, *RipD*, and *RipP1* were significantly decreased three- to fourfold under UM treatment. Finally, we observed that UM significantly reduces *R. solanacearum* population in the tobacco stem and suppresses the disease program of bacterial wilt. These results suggest that UM reduces the virulence of *R. solanacearum* by suppressing biofilm formation, transcription of the T3SS regulators and effectors. The findings indicated that UM has potential for use in the integrated control of plant bacterial wilt.

## Author Contributions

WD and LY conceived and designed the experiments. LY, XY, PL, and YZ performed the experiments. LY, WD, SL, and BL analyzed the data. WD, LY, XQ, JC, and GJ wrote and revised the paper.

## Conflict of Interest Statement

The authors declare that the research was conducted in the absence of any commercial or financial relationships that could be construed as a potential conflict of interest.

## Supplementary Material

Figure S1 The chemical structures of the tested coumarins.

Figure S2 Effect of UM on the swimming motility of *R. solanacearum*. The halo diameter of typical bacterial halos formed on semi-solid agar plates. Experiments were repeated three times, with each replicate containing at least three plates for each concentration. Statistical significance was determined by Student’s *t*-test in comparison with the DMSO control treatment.
